# Computational re-engineering of Amylin sequence with reduced amyloidogenic potential

**DOI:** 10.1186/s12900-015-0034-4

**Published:** 2015-04-24

**Authors:** Mohamed R Smaoui, Jérôme Waldispühl

**Affiliations:** School of Computer Science, McGill University, Montreal, H3A OC6 Canada; McGill Center for Bioinformatics, McGill University, Montreal, H3A OC6 Canada

**Keywords:** Mutations, Amyloid, Fibrils, Amylin, Diabetes

## Abstract

**Background:**

The aggregation of amyloid proteins into fibrils is associated with neurodegenerative diseases such as Alzheimer’s and Type II Diabetes. Different methods have explored ways to impede and inhibit amyloid aggregation. Most attempts in the literature involve applying stress to the environment around amyloids. Varying pH levels, modifying temperature, applying pressure through protein crowding and ligand docking are classical examples of these methods. However, environmental stress usually affects molecular pathways and protein functions in the cell and is challenging to construct in vivo. In this paper, we explore destabilizing amyloid proteins through the manipulation of genetic code to create beneficial substitute molecules for patients with certain deficiencies.

**Results:**

To unravel sequence mutations that destabilize amyloid fibrils yet simultaneously conserve native fold, we analyze the structural landscape of amyloid proteins and search for potential areas that could be exploited to weaken aggregation. Our tool, FibrilMutant, analyzes these regions and studies the effect of amino acid point mutations on nucleation and aggregation. This multiple objective approach impedes aggregation without stressing the cellular environment. We identified six main regions in amyloid proteins that contribute to structural stability and generated amino acid mutations to destabilize those regions. Full length fibrils were built from the mutated amyloid monomers and a dipolar-solvent model capturing the effect of dipole-dipole interactions between water and very large molecular systems to assess their aqueous stability was used to generate energy plots.

**Conclusion:**

Our results are in agreement with experimental studies and suggest novel targeted single point mutations in the Amylin protein, potentially creating a better therapeutic agent than the currently administered Pramlintide drug for diabetes patients.

**Electronic supplementary material:**

The online version of this article (doi:10.1186/s12900-015-0034-4) contains supplementary material, which is available to authorized users.

## Background

Protein misfolding has been regarded as one of the most important events triggering a wide variety of neurodegenerative and systemic diseases including Alzheimer’s, Parkinson’s, Prion disease, and Type II Diabetes [1-3]. The misfolding of certain critical soluble proteins introduces conformational changes that favor aggregation and the creation of highly ordered beta sheet rich insoluble polymers [4,5]. These structures, often referred to as amyloids in their monomeric form, or amyloid fibrils in their long aggregated form, have been observed to accumulate in the brain, heart, pancreas, and other organs. They are believed to contribute to many health problems including memory loss, brain lesions, senile plaques, synaptic spline loss, neurotic dystrophy, and cell death [6,7].

Considerable amount of work has been spent into researching ways to limit the growth of amyloid fibrils, slow their production, and inhibit their formation. Molecules have been designed to target early oligomer aggregates to prevent amyloid fibril formation [8-10]. Interestingly, others have been designed to enhance fibril formation to reduce the build up of oligomers in the cases where oligomers where toxic [11]. Other approaches included capping fibrils with docking proteins [12], inhibiting fibrils by methods of Lysine-specific molecular tweezers [13], and using sulfonated triphenyl methane derivatives as potent inhibitors [14]. Although some of these attempts have demonstrated fibril inhibition and slower reaction rates in vitro, the effect of introducing these various molecules on cellular processes, reaction pathways and other proteins is not clear and can be unfavorable.

Amyloids have been observed to undergo mutations that change their amyloidogenicity and rate formation. Several cases of Parkinson’s disease are associated with amino acid mutations of the alpha-synuclein (*α*S) protein [15-17]. The A30P mutation in *α*S decreases the overall rate of fibril formation [18,19], while the H50Q, H50A, and G51D mutants aggregate more quickly than the wild type but more slowly than A53T and E46K mutants [20]. One point mutations have been observed to be sufficient to affect the landscape of the A *β*_42_ protein in Alzheimer’s and change the internal dynamics between microstates [21]. R5A mutation studies showed a decrease both in the tendency towards A *β* aggregate formation and a reduced toxicity in Alzheimer’s [22]. A mutation in amino acid position 25 of A *β*, the loop area connecting two beta strands, has been show to destabilize A *β* fibrils [23]. Furthermore, a single mutation of serine-to-glycine at position 20 in Amylin in Chinese and Japanese populations [24] is associated with early onset of Type II Diabetes [25,26] and amplified amyloid formation [27-29]. Moreover, more than 120 single point mutations have been associated with the systemic disorderFAP [30].

As a result of the heavily observed natural mutations, research into deliberately mutating amino acids of amyloids has been proposed as an explorative method to destabilize fibrils and reduce toxicity. Computational methods coupled and steered with Molecular Dynamics runs have proved to be a viable strategy to study the impact of mutations, however, unscalable and expensive in resources and time [31]. Advancements in understanding the effects of amyloid sequence mutations on fibril toxicity and formation rate has paved the way for the development of therapeutic agents to replace highly amyloidogenic species. Re-engineering the genetic code of proteins and administering them as substitute agents for patients is a promising strategy for drug development. Pramlintide, a mutated protein version of Amylin, is used as a drug replacement in Type I Diabetes and has been shown to produce less fibrils and cause less beta cell death in the pancreas [32]. It is still unclear how point mutations alter the pathway of oligomerization and the kinetics of fibril conformational transitions [21], however, it is clear that proteins aggregate through nucleation-dependent polymerization [6,20]. Hence, exploring mutations that affect the nucleation of amyloid monomers has the potential to introduce more therapeutic agents to inhibit oligomer formation and reduce the effect of disease.

Cross-seeding of Amylin with Amylin-derived analogs has been shown to affect aggregation potential[33-35]. Although cross-seedings create mixed structures that potentially aggregate at lower rates, aggregation is inevitable due to the preservation of amino acids or amino acid regions on some chains that still favor aggregation. The idea of this work is to carefully replace amino acids in all repeating chains to create enough stress and instability to drastically lower the aggregation potential of Amylin.

In this paper we describe a protocol to analyze the structure of an amyloid protein and search for regions and residues that could be exploited to weaken aggregation. We focus our study on the diabetes-related protein, Amylin, and explore six key regions potentially contributing to amyloid oligomerization and fibril production. We developed a tool, FibrilMutant, that implements this protocol and suggests several amino acid mutations that weaken fibril structure. Subsequently, we address the multi-objective problem of discovering point mutations that destabilize fibrils yet conserve the native fold in an efficient manner by running short Molecular Dynamics simulations on single mutated native Amylin proteins to detect any initial structural turbulence and utilizing a dipolar solvent model to assess the fibril aqueous stability of much larger mutated amyloid aggregates. Our approach generated several destabilizing mutations that respect the multiple objectives. Oligomers and fibrils were built from each mutation and assessed for structural stability with an energy function that takes into account solvation energy, hydrophobicity, electrostatic interactions, and hydrogen bonding. We validate our method with results of mutations determined experimentally for Amylin and suggest new mutations that show stronger amyloid destabilizing potential than the current best therapeutic agent for diabetes.

## Methods

We created FibrilMutant to explore the effect of sequence mutations on destabilizing amyloid fibrils. FibrilMutant takes a Protein Data Bank (PDB) file of a single amyloid monomer and a PDB file of its native protein conformation as input and generates single point mutations to destabilize the protein’s fibril structure. We apply the mutations to both the native and the amyloid form of the protein and calculate the stability effect on both forms. The following procedure is followed to generate and assess the mutations:

1. Load the PDB file into FibrilMutant and analyze key structural characteristics contributing to aggregation.
2. Explore a set of mutations that could weaken regions contributing to aggregation.
3. Generate fibril stability landscapes to find the most stable fibril polymorph of the amyloid PDB monomer.
4. Build mutated fibrils of the most stable polymorph.
5. Assess the stability of each mutated fibril in water with a dipolar water modal.
6. Discard any mutation that stabilizes the amyloid fibrils, and verify with short full Molecular Dynamics (MD) simulations that the final mutation list does not introduce structural lump turbulences that destabilize the native protein.

FibrilMutant builds on our recent work that enabled the simulation of accurate fibril models [36]. Its core development includes predicting a set of effective mutations and building oligomers and large fibrils to test the effects of these mutations on structural stability. A detailed description of the procedure is described below.

### Step 1: Analyzing amyloid structures

Amyloids share key structural similarities. FibrilMutant extracts from a PDB file regions with beta strands, screens regions at beta turns, identifies salt bridges, and examines hydrophobic, polar and charged residues. This collected data is then utilized to generate amino acid mutations in the extracted key regions and residues. The protocol followed by the algorithm in choosing destabilizing mutations is outlined in Table 1. In short, for each extracted region in the amyloid, the algorithm selects mutations that lower the stability contribution of that region to the overall amyloid structure. For example, beta turns contribute to amyloid stability by providing the needed torsional flexibility for beta sheets to form. To destabilize the beta turn regions of the amyloid, the algorithm mutates amino acids on the beta turns into clunky prolines, causing torsional stress and extending the torsional angles in the turns. This causes beta strands to drift apart and weakens beta sheet bindings. Another example is salt bridges. Some amyloids contain salt bridges that strongly stabilize their 3-dimensional forms. If the algorithm detects a salt bridge, it attempts to break it by mutating one of the amino acids that contributes to the salt bridge into a non-charged, non-polar residue. Based on our observation regarding amyloid structure, we outlined in Table 1 six stability regions in amyloids that could be exploited to destabilize structure. In Table 2 we show the specific residues of Amylin that belong to each stability region.

**Table 1:** **Effect of mutation choice on structural stability**

**Structure characteristic**	**Contribution to amyloid stability**	**Disruption method**
Hydrophobic Core	Hides core residues from water and generates a packed core	Mutate a hydrophobic residue in the core into a charged one
Hydrophilic Surface	Provides a stable contact surface to water	Mutate a polar residue on the surface into a hydrophobic one
Beta Sheets	Constitute the backbone of fibrils	Decrease the number of hydrogen bonds
		between Beta strands
Beta Turns	Provide needed torsional flexibility for Beta sheets to form	Mutate the center residue and any Glycine amino acid of a Beta turn region into a Proline to limit torsional flexibility
Salt Bridges	Produce an ionic bond between fibril monomersor the monomer itself	Search the amyloid structure than 4.5 Å apart bonding the following for bonds less pair of amino acids: ASP - LYS, ASP - ARG, GLU - LYS, GLU - ARG, and mutate one amino acid into a non charged, non polar residue to break the ionic bond.
Polar Regions	Contribute hydrogen bonds	Mutate polar residues into non polar ones to weaken hydrogen bonds

This table summarizes our approach to choosing mutations to test for fibril destabilization. We identify six main features of amyloids that contribute to structure stability and outline the methods we used to weaken their contribution to the amyloidogenicity of proteins.

**Table 2:** **Structural stability regions of Amylin**

**Structure**	**Sequence region**
**characteristic**	**1———–9———–18———–27———–37**
Hydrophobic Core	—————LA-FLV——-FGAI—————
Hydrophilic	-CNT-TC-TQ————————————-
Surface	
Beta Sheets	——TCATQRLANFLVH——-AILSSTNVGSNT-
Beta Turns	————————–SSNNFG——————
Salt Bridges	—————————————————-
Polar Regions	-CNT-TC-TQ—-N—-HSSNN——SSTN–SNTY
Charged residues	K————-R————————————

This table outlines the residues that belong to each stability region in Amylin.

### Step 2: Generating mutations

Structural data collected from an amyloid PDB file is used to predict destabilizing mutations. The inner core of amyloids is known to be a hydrophobic core. One way to disrupt this core is by introducing a mutation of one of its amino acids into a charged residue. We have explored several ways mutations can weaken the structure stability of amyloids. Upon selection of potential mutations, we run TANGO [37], a tool to estimate aggregation propensity using a statistical mechanics algorithm, to quickly rank candidate mutations and provide preliminary data on the potential destabilizing effect of each mutation. We understand that TANGO uses a coarse-grained model to perform high-throughput screenings of results, hence we only use it as a guide to initially rank the numerous mutations.

### Step 3: Fibril stability landscape

Amylin has recently been found to form into a fibril structure composed of two stacked protofibrils [38]. However, the experimental structural parameters, mainly fibril rotation angle and protofibril packing distance, have not been published yet. MD simulations could be used to find these parameters but the process is computationally expensive in resources and time, and we estimated these by our previous work on Stability Landscapes [36].

Starting with an accurate crystal or NMR amyloid monomer, we first define a range of naturally possible values for the various fibril degrees of freedom characterized by rotation angles, packing distances and beta strand proximities. Second, we utilize these range of values to construct all possible fibril structures using rigid affine transformations. Third, we perform light runs of Energy Minimization (a few hundred steps of relaxing the protein structure and removing close clashing atoms) on each generated structure to assess its initial stability sensitivity by calculating any enthalpy drift between final and initial conformations. This step creates the fibril stability landscape by exhausting all suitable “parameter” values. We then search the landscape for values that construct the most stable initial conformation. These “parameter” values would create structures with lowest enthalpy drift and lowest initial Lennard-Jones and Coulomb terms. Structures with low enthalpy drifts allude to stable conformations (local minima on the structural energy landscape of fibrils), and structures with high energy drifts suggest parameters that produce unstable conformations.

### Step 4: Building fibril models

A different amyloid monomer is generated for every mutation. To test the stability effect of the mutations on fibrils, these mutated amyloid monomers need to assemble into fibrils. Fibrils are polymorphic and since we don’t know the polymorph this specific amyloid protein will aggregate in, we first need to figure out which polymorph is the most stable for the current protein. To do this, we resort to our previous work, CreateFibril, a tool that builds and explores the stability of fibrils. Once we know the specific polymorph the protein will aggregate in, we apply mutations to the amyloid monomer and construct a new mutated structure with SCWRL [39]. SCWRL is a tool that determines side-chain conformations to a backbone structure. We specified the original backbone of the protein, but gave SCWRL the mutated sequence of amino acid to fit onto the structure. We then perform energy minimization on the mutated structures to remove any steric clashes due to mutations. The structure is then built into the correct fibril polymorph by CreateFibril.

### Step 5: Assessing mutated fibril structural stability

Applying point mutations to a structure can introduce steric clashes between amino acid side chains. To combat this issue, we perform Energy Minimization (EM) to relax all mutated amyloid and native structures. After this process of relaxation, we use our previous work to quickly calculate the Free energy of proteins given by Eq. (1). We calculate the LJ and Coulomb energies and use a fast and detailed dipolar water model to compute the solvation energy of molecules by solving the dipolar nonlinear Poisson-Boltzmann-Langevin equation. Together, the three energy terms are used to describe the stability behavior of fibrils. Fibrils with higher energy than the natural control are termed as unstable, and the mutations that generate them are kept for further analysis on the native structure. We used the program AquaSol [40] with the following setup: atomic charges and radii assigned with PDB2PQR using CHARMM force field at neutral pH. A grid or 257 points per edge spaced by 1 Å, a temperature of 300K, and a solvent accessible surface with an Rprobe of 1.4 Å. All hydrogen-bonds were optimized. We used a trilinear interpolation protocol for projection of fixed charges on the grid, a lattice grid size for the solvent:a = 2.8 Å, solvent made of dipoles of moment *p*_0_= 3.00D at a concentration of *C*_*dip*_= 55M. No salt was added to the solution and small ions were used to equilibrate the system when needed. The electrostatic potential was set to zero at the boundaries, and the stopping criteria for residual was sent to: 1.10^−6^ (when possible).

(1) $$\begin{array}{@{}rcl@{}}  F_{E} &= F_{solv} + F_{coulomb14} + F_{vdw} \end{array} $$

where,

$$\begin{array}{@{}rcl@{}} F_{solv} &=& F_{(p_{0}, C_{dip})} - F_{(0, 0)} - N_{w}\mu_{w} \\ \mu_{w} &=& k_{B} T \frac{ln\left(1-N_{A} C_{dip} a^{3}\right)}{N_{A} C_{dip} a^{3}} \\ N_{w} &=& \int_{solvent} d \mathbf{r} \rho_{dip} (\mathbf{r}) \\ \end{array} $$

### Step 6: Structural deviations of the native protein

This step is crucial in addressing the multi-objective problem of discovering point mutations that destabilize fibrils yet conserve the native fold. Mutations that create unstable fibrils are applied to the native protein form to assess any structural stability effect they could produce. We are interested in mutations that destabilize the amyloid but not the native form. Such mutations theoretically preserve structure and protein function and are candidates for therapeutic engineering. We use SCWRL to build the mutated native proteins and run Energy Minimization (EM) to relax the structures. We then run a full MD simulation and plot RMSD and RMSF graphs to verify any structure deviations caused by the mutation. In particular, we calculate the perturbation in structural motion with

$$\begin{array}{@{}rcl@{}} \delta_{rmsd} = RMSD(mutant) - RMSD(native) \end{array} $$

where, *RMSD* measures the root mean-square deviations, in angstroms, of the *C*_*α*_ atom positions in a protein’s residues over a simulation run.

We also calculate the root mean square fluctuations (RMSF), a measure of the deviation between the position of a particle *i* over a simulation run,

$$\begin{array}{@{}rcl@{}} RMSF = \frac{1}{T} \sum_{t_{j} = 1}^{T}{ \left(x_{i}\left(t_{j}\right) - \tilde{x_{i}} \right)^{2} } \end{array} $$

where *T* is the total simulation time, and $\tilde {x_{i}}$ is the reference position of particle *i*. Applied to our Amylin protein, the reference positions are the 37 amino acid residues of the protein. The *RMSF* value at each residue measures the residue’s average change in position over the simulation run. Low *RMSF* values at a particular mutation site suggests the absence of local residualinstability.

### Molecular dynamics and energy minimization

We used the GROMACS 4.5 [41] molecular simulation package to run molecular dynamics (MD) and EM simulations. Our mutated proteins were solvated in a cubic box (with a minimum distance of 35 Å from any edge of the box to any atom) and neutralized with chloride ions and modeled using the GROMOS96 53a6 force field along with the SPC water model. This force field is designed for bimolecular dynamics simulations in MD productions and handles protein structures very well [42,43]. The force field reproduces the free enthalpies of hydration and apolar solvation for a range of compounds, including amyloid proteins. We use this force field in studies involving amyloid structures and amyloidogenicity potentials. We used a cutoff of 10 Å for van der Waals and short range electrostatic interactions, and calculated long range electrostatic interactions using a particle mesh Ewald sum [44,45]. Simulations were prepared for a full MD run in both isothermal-isobaric [46] (100 ps) and canonical equilibration (100 ps) ensembles. Temperature and pressure were controlled at 300 K and 1 bar using the velocity rescaling thermostat and the Parrinello-Rahman barostat, respectively. A linear constraint solver was used to keep all bonds at their equilibrium length. One million time steps were used with an integration time step of 2 fs. The system’s coordinates were saved every 10 ps for furtheranalysis.

### Analyzing energy results

To assess the effect of mutations on amyloid fibril stability, we generated fibril mutants up to 25 monomers in size and used Eq. (1) to calculate their energies. The solvation term was calculated by AquaSol while the LJ and Coulomb terms were calculated by GROMACS. The same formula was used in the initial assessment of the mutated native structures. We generated RMSD and RMSF plots from MD simulations to analyze structural changes and residue perturbations in native Amylinmutants.

## Results and discussion

In this section we apply our methods to the protein Amylin (PDBID 2KB8 [47]), a 37 residue peptide hormone that is secreted from the pancreas in response to intake of food. The 2KB8 is a micelle-stabilized NMR structure suited for diabetes protein-membrane aggregation studies. This structure is the non-amyloid form of amylin that misfolds into amyloids. We refer to this structure as the “native” form of Amylin throughout the remaining of the paper. Amylin normally contributes to glycemic control and inhibits the appearance of specific nutrients in the plasma [48,49]. In patients with Type II Diabetes, Amylin has been found to misfold into destructive amyloid monomers that aggregate in pancreatic beta cells and disturb cellular activity, disrupt flow of ions through membranes, and force cells to apoptosis [50,51]. Little is known about the mechanism or pathway behind the misfolding event, however, the structure of Amylin’s amyloid protein is known. Patients with Type I Diabetes are unable to produce Amylin in their pancreas and require Amylin injections. In 2005, Pramlintide, a version of Amylin with three point mutations that has a lower affinity to form amyloids and fibrils was introduced in the treatment of Type I and Type II Diabetes [52], and has been a better substitute for Amylin in patients with diabetes. Pramlintide, however, is not optimal as patients still experience the emergence of some fibrils that further destroy their *β*-cells. In this section, we present the results of applying our FibrilMutant protocol on analyzing Amylin’s conformational regions and stability landscape. Moreover, we show that our energy function and destabilizing criteria are in agreement with experimentally tested Amylin mutations and discover novel mutations with stronger destabilizing potential and lower fibril affinities thanPramlintide.

### Exploring key stability regions of Amylin

**Figure 1:**
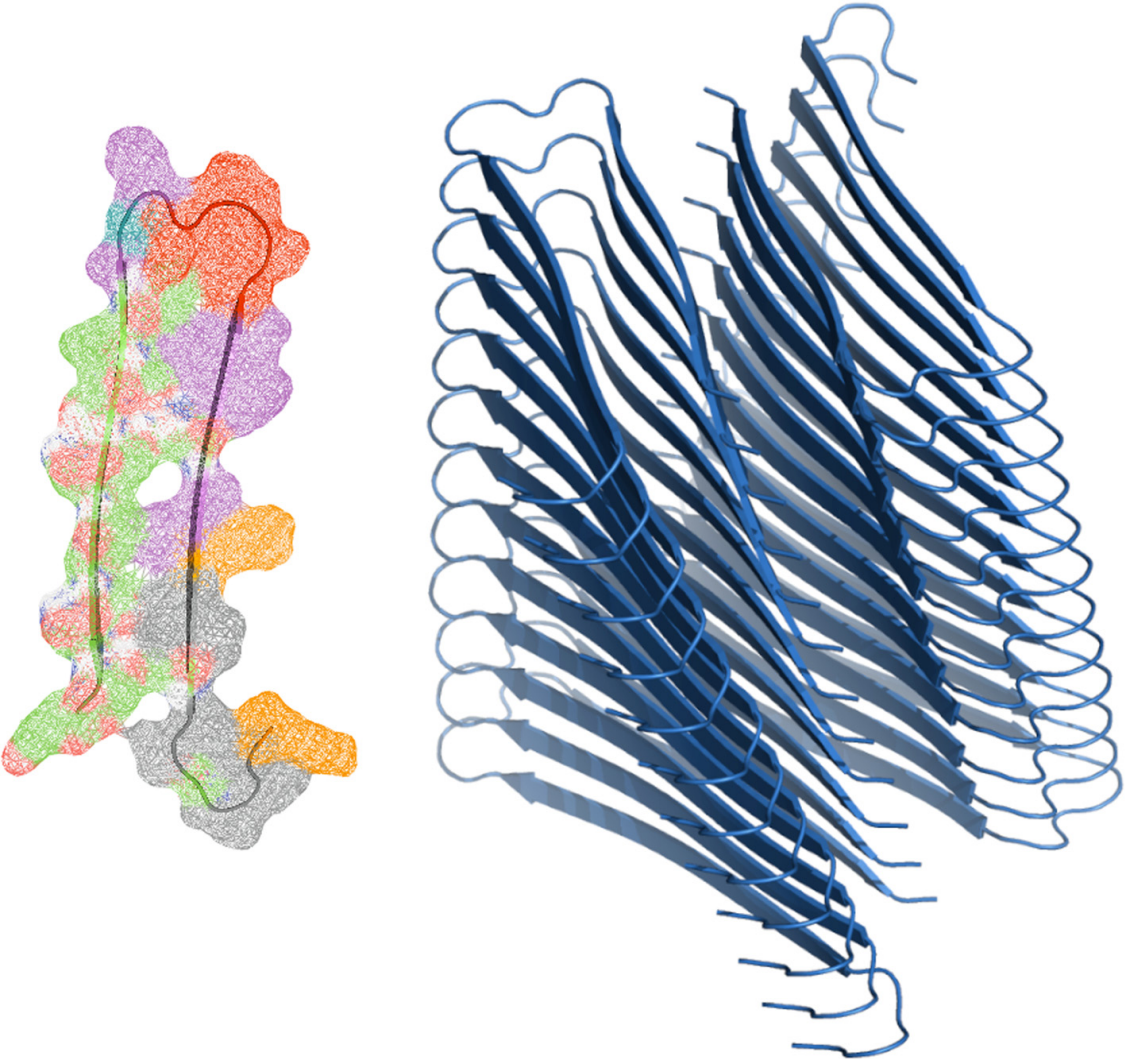
Amylin amyloids. Left: Identification of key stability regions of amyloid Amylin by FibrilMutant. Beta strands are colored green, beta turns red, charged residues orange, hydrophobic residues purple, polar residues grey, and glycine residues at turns blue. Right: Full Amylin fibrils of 25 monomers in size.

The protocol we implemented into FibrilMutant identified six key stability regions in the amyloid form of Amylin that contribute to the emergence of amyloids and the growth of their fibrils, as shown in Table 2. We used the PDB models devised by Wiltzius et al. from experimental data [38] that aggregate into the only observed 2-Stack structures. FibrilMutant generated twenty three single point mutations with potential to destabilize Amylin fibrils, possibly hindering their production or slowing down their aggregation. The mutations were initially ranked by a statistical mechanics algorithm used in TANGO [37] to help us prioritize simulation order. Table 3 displays these mutations. These suggested mutations imply that Amylin amyloid fibrils are stabilized by the following four main factors: a hydrophilic surface in contact with water, a large hydrophobic core region, beta strands, and glycine amino acids at beta sheet turns as illustrated in Figure 1. All proposed mutations attempt to destabilize these regions to weaken Amylin fibril structures.

**Table 3:** **Amylin Mutations generated by FibrilMutant with destabilizing potential**

**Original**	**Residue no.**	**Mutated**	**Disruption**	**TANGO**
**amino acid**		**amino acid**	**method**	**rank**
A	13	R	Making core charged	1
F	15	P	Mutating an amino acid on a beta strand	2
F	15	D	Making core charged	3
L	16	D	Making core charged	4
A	25	R	Making core charged	5
I	26	R	Making core charged	6
G	24	P	Mutating GLY at a turn	7
L	27	R	Making an amino acid on a beta strand charged	8
F	23	E	Making core charged	9
G	24	D	Making core charged	10
V	17	E	Making core charged	11
Q	10	H	Making protein surface hydrophobic	12
N	21	P	Mutating an amino acid at a turn	14
C	2	Q	Making protein surface hydrophobic	15
T	6	M	Making protein surface hydrophobic	16
T	4	S	Making protein surface hydrophobic	17
V	32	K	Making an amino acid on a beta strand charged	18
N	3	H	Making protein surface hydrophobic	19
A	8	E	Making an amino acid on a beta strand charged	20
T	9	N	Making protein surface hydrophobic	21
L	12	E	making core charged	23
C	7	T	Making protein surface hydrophobic	24
G	33	E	Making an amino acid on a beta strand charged	25
S	20	G	Discovered experimentally [29]	13
S	20	K	Discovered experimentally [29]	22
N	21	L	Discovered experimentally [56]	26
N	14	L	Discovered experimentally [56]	27

Mutations above the horizontal line are destabilizing mutations proposed by FibrilMutant, and mutations below the line have been suggested and tested experimentally. Mutations are ranked by TANGO from lowest aggregation potential to highest aggregation potential.

### Generating Amylin fibrils

To test the effectiveness of the mutations suggested by FibrilMutant, we apply the mutations to Amylin fibrils ranging in size from one to twenty five monomers as shown in Figure 1. Amylin fibrils have been found to form into a dimer conformation recently classified as a 2-Stack [38]. Using fibril stability landscapes from our previous work [36], we construct the mutated structures with a fibril packing distance of 3.0 Å, Hbond distance of 5.0 Å between monomers and a rotation angle of 9 degrees along the fibril axis. Each fibril contains one amino acid mutation on each of its amyloid monomers. The stability landscapes are efficient-exhaustive search heuristics for structural parameters that create energetically optimal fibril shapes.

### Analyzing Amylin fibrils

**Figure 2:**
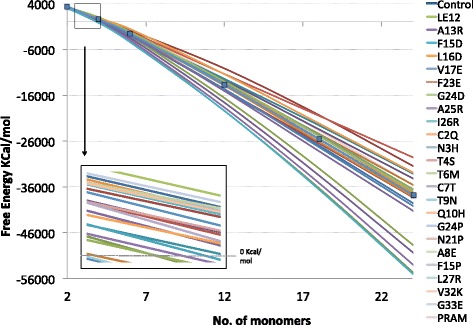
Free energies of Amylin mutated fibrils calculated with Eq.1. The inner box is a close up showing energies at the nucleation phase. The control wild type fibril is marked with boxes along its curve. All fibrils with lines above the control are less stable than the control, and all fibrils below the control increase fibril stability.

Figure 2 shows the free energies of the most significant fibrils at their nucleation phase and at their extended aggregation phase. Energy values higher than the control fibril correspond to a decrease in stability, while lower energies correspond to an increase in fibril stability. We are interested in mutations that create the most instability. To be able to sort out these better mutations, we developed three formulas to rank the mutations according to the metrics that measure a protein amyloidogenicity factor, a fibril nucleation factor, and a fibril aggregation extension potential factor. Together, these metrics are intended to measure stability deviations in the various stages of fibril development and growth. We measure the amyloidogenicity factor by

(2) $$\begin{array}{@{}rcl@{}} \Delta G^{i} = F^{i}_{a_{1}} - F_{n_{1}} \end{array} $$

where *ΔG*^*i*^ is the free energy resulting from transforming an Amylin protein from a mutated native to a mutated amyloid fold, $F^{i}_{a_{1}}$ is the free energy of a single amyloid monomer and $F_{n_{1}}$ is the free energy of a single native protein for all mutated fibrils *i*.

The second metric is

(3) $$\begin{array}{@{}rcl@{}} \Delta N^ i = F^{i}_{a_{4}} - 4~ F^{i}_{a_{1}} \end{array} $$

where *ΔN*^*i*^ is the free energy of nucleation resulting from joining four free amyloid monomers into a fibril structure, $F^{i}_{a_{4}}$ is the free energy of an Amylin fibril composed of 4 monomers and $F^{i}_{a_{1}}$ is the free energy of a single amyloid monomer for all mutated fibrils *i*.

The third metric, $\Delta \tilde {F}^{i}$, measures the difference in energy between the mutated fibril and the control averaged out over the length of the fibril,

(4) $$\begin{array}{@{}rcl@{}}  \Delta \tilde{F}^{i} = {\sum_{j}^{n}} {\frac{F^{i}_{a_{j}} - F^{c}_{a_{j}}}{j} } \end{array} $$

where $F^{i}_{a_{j}}$ is the free energy of the mutated fibril *i* at length *j* and $F^{c}_{a_{j}}$ is the free energy of the control wild type fibril at length *j*.

**Figure 3:**
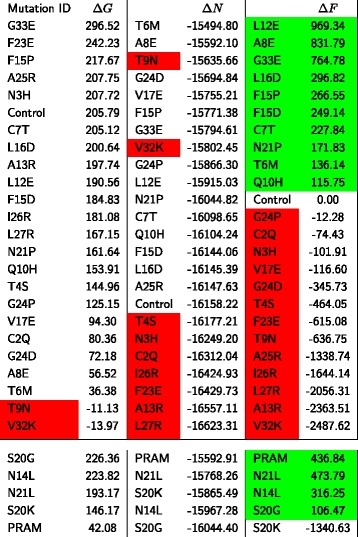
Stability values of mutated fibrils. Mutations above the horizontal line were proposed by FibrilMutant. Mutations below the line were explored experimentally. The “Control” fibril is the non-mutated, naturally occurring fibril. Units are in KCal/mol.

Together, *ΔG*,*ΔN* and $\Delta \tilde {F}$ provide insights into the stability perturbations caused by the mutations at the amyloid formation phase, fibril nucleation phase, and fibril elongation phase, respectively. Figure 3 shows the energy values of all the three metrics applied to the mutated fibrils. A positive *ΔG* value describes an endothermic reaction where native Amylin structures required an input of energy to form into amyloid monomers. The higher the *ΔG*, the higher the gap in energy between amyloids and native Amylin. Negative *ΔG* suggest favorable exothermic reactions and possibly spontaneous formation of fibrils. Hence, mutations in Figure 3 with negative *ΔG* are eliminated in red.

*ΔN* is useful in comparing the strength of fibrils created at nucleation. The more negative *ΔN* values produce stronger exothermic reactions, and hence more stable fibrils. For our study, we want to explore the mutations that produce a *ΔN*^*i*^ greater than *ΔN*^*c*^ (control). We rank the mutations in the middle column of Figure 3 from weakest to highest and remove all mutations smaller than *ΔN*^*c*^. Finally, $\Delta \tilde {F}^{i}$ estimates the stability deviation of the mutated fibril from the control wild type. Positive values suggest fibrils that are weaker than the control and negative values suggest fibrils more stable than the control. We observe that the energy gap widens between fibrils and the control as fibrils grow in size which suggests that unstable fibrils (high energy difference with respect to the control) are likely to create energetically preferred shorter structures, possibly a better chance for degrading enzymes and macrophages to destruct them [53,54]. We ranked the filtered mutations in Figure 3 and highlighted in green the unstable fibrils out of our set.

The sensitivity of amyloid formation to point mutations can be exploited to design slower and shorter Amylin aggregating variants which cells might be able to discard. There has been no reported systematic analysis of all of the amino acid positions of IAPP or their amyloidogenicity potential, and mutation studies are sparse [55]. We validate our method and rankings by first considering the few mutations explored experimentally [29,56]. Amylin N14L and N21L mutants did not form amyloids experimentally while, the S20K mutant lengthened the lag phase by a factor of 18 and had a significant effect on amyloid formation and S20G was observed to form amyloids. The lower part of Figure 3 shows the *ΔG*, *ΔN*, and $\Delta \tilde {F}$ of these 4 mutations. We observe that N14L and N21 have a higher *ΔN* and $\Delta \tilde {F}$ than the control, suggesting that these mutations destabilize their fibrils, and hence could explain why they do not form experimentally. S20K has a *ΔN* that is also higher than the control, suggesting that the nucleation product is less stable than the control’s oligomer, also suggesting a longer nucleation phase as observed experimentally. The S20K $\Delta \tilde {F}$ is quite small, suggesting that this mutation might form unstable fibrils, as observed experimentally. The S20G mutant was observed to form amyloids, and its corresponding $\Delta \tilde {F}$ also suggests this finding. It is key to note that Pramlintide ranked as the highest unstable mutant explored experimentally with a $\Delta \tilde {F} = 436.84$, close in instability to the N21L mutant.

Our results indicate that the mutant L12E causes the most instability to fibrils and has a high *ΔG*, *ΔN*, and $\Delta \tilde {F}$. In fact, its $\Delta \tilde {F}$ is more than twice as large as the PRAM $\Delta \tilde {F}$, suggesting that it might inhibit fibrils altogether. The mutant A8E also has twice as large a $\Delta \tilde {F}$ than PRAM, but also has a slower more unstable nucleation phase, indicating that it has a strong potential to inhibit fibril formation. In fact, the results of running AmyloidMutants [57] on the A8E mutation suggest that this point mutation destabilizes amylin fibrils, as shown in Additional file 1: Figure S1. The last competitive mutant, G33E, also exhibits a higher $\Delta \tilde {F}$ than Pramlintide and shows a high *ΔN* value, also suggesting high instability in the nucleation phase and fibril elongation phase. Together, these observations recommend a Glutamic acid mutation in Amylin to stop it from forming fibrils. Since Amylin contains no acidic residues, the addition of this charged, acidic residue will enhance the formation of a quasi-infinite array that destabilized the fibrils with unfavorable electrostatic interactions created along the fibril length [29]. The other highlighted mutations in green in Figure 3 also have the potential to destabilize and inhibit fibrils, and their effect might be similar or smaller than Pramlinitide. Although the energy plots in Figure 2 suggest that destabilizing mutations continue to show an increase in fibril stability as aggregation increases, this doesn’t necessarily mean that aggregation will happen. The instability introduced by the mutations could increase the activation energy beyond the physiological means required for misfolding and aggregation to occur. An excellent example is rat Amylin; it contains three mutations that increase the instability of its amyloid fibrils and hinder it form aggregating in vivo. However, under the right environment conditions, the aggregation of rat Amylin into long fibrils can still occur [58]. Nevertheless, the three destabilizing mutations introduced enough instability to create an activation barrier that is difficult to surpass in physiological conditions. The results we report in this study are important to design stronger alternative variants to the Pramlintide antihyperglycemic drug with a minimalistic mutation approach for diabetes patients.

TANGO and AmyloidMutants are current computational tools exploring amyloid stability and analyzing the effect of secondary structure modifications on increasing amyloidogenicity and protein aggregation. Such tools use coarse-grained models that enable them to perform high-throughput screenings, but cannot achieve the accuracy of higher resolution models [31]. The use of AmyloidMutants and TANGO assisted in ranking candidate destabilizing mutations prior to running our computationally expensive dipolar solvent model to accurately assess the instability caused in the mutated fibrils. Although the TANGO and AmyloidMutants results did not match well with our results, they did provide some valuable insight. TANGO results in Additional file 1: Table S1 suggest that the A8E mutation doubles the potential of alpha helices compared to the Control and the L12E mutation lowers the amyloidogenicity by a factor of 42 compared to the Control. AmyloidMutants also suggested that the A8E mutation should destabilizefibrils.

### Maintaining native structure & function

**Figure 4:**
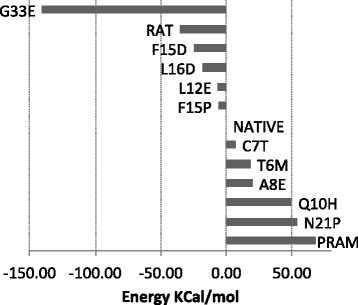
Stability of the Amylin mutants in their native fold. Each bar represents one mutated protein in native fold, where the energies are the free energy differences between the mutant and the non-mutated Amylin.

**Figure 5:**
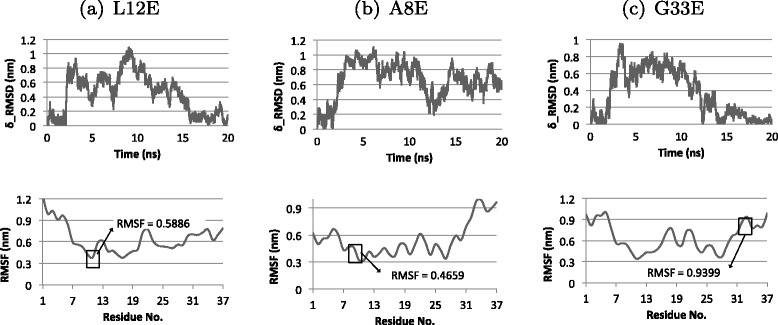
*δ*_*rmsd*_ and RMSF plots for mutants L12E, A8E, and G33E over a 20ns simulation. The top figures display *δ*_*rmsd*_ graphs between each mutant and the native Amylin, and the bottom plots are the RMSF plots. **(a)** Mutant L12E shows a *δ*_*rmsd*_<1.2 and an RMSF <0.6 at residue 12, **(b)** mutant A8E exhibits a *δ*_*rmsd*_<1.1 and RMSF <0.5 at residue 8, and **(c)** mutant G33E has a *δ*_*rmsd*_<1 and an RMSF <1 at residue 33. *δ*_*rmsd*_ and RMSF results show extremely small modifications in structure and minimal structural variance at the amino acid mutation sites.

Introducing a mutation to the sequence of Amylin might cause a change in its structure and force it to fold into a different shape, affecting its normal function. For this reason, we have tested the effect of the destabilizing mutations on the native structure of Amylin. The free energies of mutated Amylin in Figure 4 show that the mutations result in energy states close to the native control, rat Amylin, and pramlintide. Figure 4 suggests that these mutations do not make native Amylin unstable. To further verify this, we ran 20 ns MD simulations on the top 3 native mutants (L12E, A8E, and G33E) to test any structural turbulence or major energetic imbalances as a result of the point wise substitutions. We observed very slight deviations in *δ*_*rmsd*_, stable RMSF in mutated regions and smooth energy trajectories, suggesting a preservation in shape and function. Figure 5 shows the *δ*_*rmsd*_ and RMSF results for best mutants, L12E, A8E, and G33E. Although the simulation time that was used does not provide a definitive conclusion on the stabilities of the three mutants, the *δ*_*rmsd*_ and RMSF results point towards native confirmation stability.

## Conclusion

The process of amyloid protein formation and aggregation is sensitive to amino acid sequence point mutations. We discuss in this manuscript how altering the genetic code of these amyloid proteins has been shown to affect fibril stability, monomer propagation and growth (see Figures 2, 3 and 4). The RMSD and RMSF molecular dynamics results in Figure 5 simulate the change in the shape of the mutant amyloid structures during the process of aggregation, which can have a direct effect on the infectivity and toxicity of fibrils. The less stable a fibril structure becomes, the higher the potential for a decrease in toxicity. Certain regions in amyloid proteins contribute to fibril structural stability, compactness, and insolubility. Altering some amino acids that make up these regions, such as the amino acids that are involved in creating a hydrophobic core, can create energy perturbations and imbalances that weaken an individual amyloid protein monomer and subsequently carry on the effect to weaken every amyloid monomer on the fibril, resulting in an accumulated fibril destabilization effect. Destabilizing one amyloid form can potentially lead it to aggregate into a different form. Since the current Amylin form is a 2-Stack (dimer amyloid), we assume in this work that the destabilization would weaken the 2-Stack structure. It is possible that the 2-Stack separates into single linear amyloid aggregates. However, it is unlikely that the destabilized 2-Stacks form into tri-mers (3 connected linear strands). For this reason, it is important to examine the resulting destabilizing set of mutations and experimentally select those that cause instability to the core of the protein, rather than causing instability on the contact surface holding 2-Stack structures together. Tackling the problem from this perspective has enabled us to computationally perform an amino acid mutation analysis on Amylin to unravel modifications that potentially destabilize fibrils, yet are restricted to conserve the native fold of the protein. Addressing this multi-objective problem can be generally useful in suggesting novel therapeutic agents or improving existing treatments for cases where drugs have to be administered to patients. In our case, addressing this problem has opened up discussions on the 3 potential efficacy improvements in the Pramlintide drug for Diabetes.

## Additional file

Additional file 1 **Supplementary material.** **Figure S1.** presenting a simulation of AmyloidMutant on the 3 mutations. **Table S1.** showing a run of TANGO of the mutations we tested.

## Abbreviations

*α*S: alpha-synuclein protein
PDB: Protein Data Bank
LJ: Lennard-Jones
EM: Energy Minimization
MD: Molecular dynamics
NMR: Nuclear Magnetic Resonance spectroscopy
RMSD: Root-mean-square deviation
RMSF: Root-mean-square fluctuation
