# Supplementary material for: Computational re-engineering of Amylin sequence with reduced amyloidogenic potential
Source: BMC Struct Biol. 2015 Apr 24;15:7. doi: 10.1186/s12900-015-0034-4 (PMC4428086; doi:10.1186/s12900-015-0034-4)
Supplement: Supplementary file 1 — Supplementary material. Figure S1. presenting a simulation of AmyloidMutant on the 3 mutations. Table S1. showing a run of TANGO of the mutations we tested. [file 12900_2015_34_MOESM1_ESM.pdf]

Supporting Information

1 Results

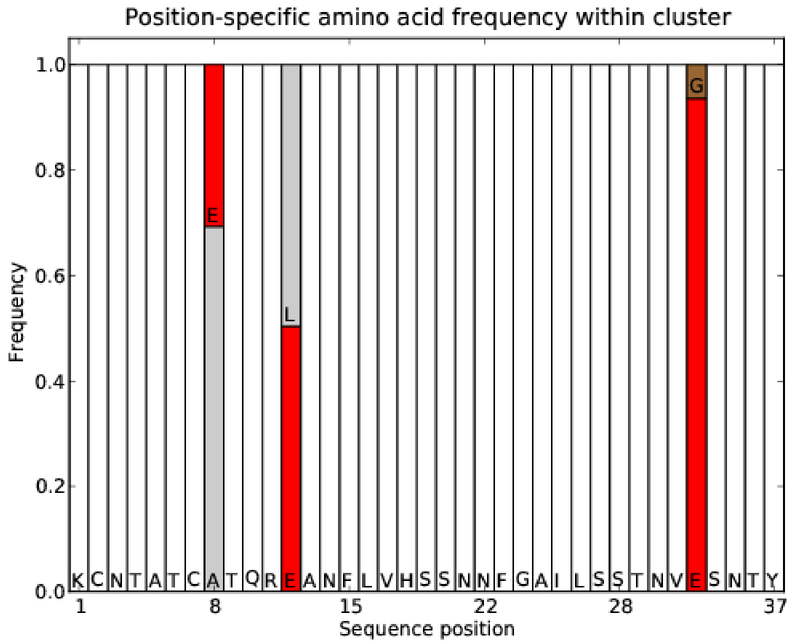

Figure S1: Top 3 mutation results from AmyloidMutants. Mutation A8E presents a high destabilization fibril aggregation frequency.

TANGO Rank	Mutation	AGG	AMYLO	TURN	HELIX	BETA
1	A13R	17.4231	0.00229692	26.2032	46.2422	50.1558
2	F15P	17.4231	0.00483631	24.9158	50.5216	44.2985
3	F15D	18.6542	0.00494376	26.6371	18.5491	45.443
4	L16D	18.6542	5086.05	27.7912	26.473	55.8228
5	A25R	19.9643	11265.8	27.9887	38.6424	55.1474
6	I26R	19.9643	11265.8	27.6605	39.8376	43.7964
7	G24P	19.9643	11265.8	25.3545	38.6424	47.8267
8	L27R	19.9643	11265.8	27.6375	38.6424	47.5071
9	F23E	20.1387	11265.3	28.4565	42.7176	46.663
10	G24D	20.1387	11265.3	26.3146	40.1409	52.1159
11	V17E	27.6387	11303.4	27.8741	37.1066	42.0603
12	Q10H	36.4611	11348.7	27.7108	4.22761	52.4841
14	N21P	37.2516	11271.1	39.9629	41.3196	47.075
15	C2Q	37.3071	11266.1	27.5314	41.2988	46.1128
16	T6M	37.311	3600.44	26.6308	44.1818	41.6555
17	T4S	37.3528	12404.1	27.732	43.2968	47.3828
18	V32K	37.3697	11265.8	29.2945	41.3929	45.9617
19	Control	37.3874	11265.8	27.3615	41.4001	47.7778
20	N3H	37.4431	13070.4	27.169	37.9999	51.2714
21	A8E	37.4799	12261.2	26.8771	92.3333	53.2163
22	T9N	37.5127	12620.4	28.8211	33.0901	45.0847
24	L12E	37.6587	264.028	25.8306	23.7728	48.1199
25	C7T	37.6787	4923.45	26.176	26.4939	47.923
26	G33E	38.7927	11265.3	21.6298	41.3269	64.2312
13	S20G	36.959	11266.7	31.3738	41.3718	47.3916 [29]
23	S20K	37.5908	11268.8	24.84	39.3714	49.6538 [29]
27	N21L	42.4584	11266.8	20.5032	42.4313	61.4794 [54]
28	N14L	503.96	12778.5	24.8149	23.0706	51.5248 [54]

Table S1: TANGO results on amylin mutations. Mutations above the horizontal line are destabilizing mutations proposed by FibrilMutant, and mutations below the line have been suggested and tested experimentally. Mutations are ranked by TANGO from lowest AGG potential to highest AGG potential. The TANGO algorithm predicts cross-beta aggregation in proteins and populates structures according to a Boltzmann distribution to 4 structural stats: beta-sheet aggregation, beta-turn, alpha-helix, and alpha-helical aggregation. The results in this table describe the following in respective order: percentage of aggregation, amyloidogenicity potential, percentage of beta-turn conformation, percentage of alpha-helical conformation, and percentage of beta-strand conformation
